# Sphingosine-1-phosphate and CRP as potential combination biomarkers in discrimination of COPD with community-acquired pneumonia and acute exacerbation of COPD

**DOI:** 10.1186/s12931-022-01991-1

**Published:** 2022-03-20

**Authors:** Chin-Wang Hsu, Chi-Won Suk, Yuan-Pin Hsu, Jer-Hwa Chang, Chung-Te Liu, Shau-Ku Huang, Shih-Chang Hsu

**Affiliations:** 1 Emergency Department, Wan Fang Hospital, Taipei Medical University, Taipei, Taiwan; 2 Department of Emergency, School of Medicine, College of Medicine, Taipei Medical University, Taipei, Taiwan; 3 Division of Pulmonary Medicine, Department of Internal Medicine, Wan Fang Hospital, Taipei Medical University, Taipei, Taiwan; 4 Graduate Institute of Clinical Medicine, College of Medicine, Taipei Medical University, Taipei, Taiwan; 5 School of Respiratory Therapy, College of Medicine, Taipei Medical University, Taipei, Taiwan; 6 Division of Nephrology, Department of Internal Medicine, Wan Fang Hospital, Taipei Medical University, Taipei, Taiwan; 7 Department of Internal Medicine, School of Medicine, College of Medicine, Taipei Medical University, Taipei, Taiwan; 8 National Institute of Environmental Health Sciences, National Health Research Institutes, Zhunan, Taiwan; 9 Lou-Hu Hospital, Shen-Zhen University, Shen-Zhen, China; 10 Research Center for Environmental Medicine, Kaohsiung Medical University, Kaohsiung, Taiwan; 11 Johns Hopkins Asthma and Allergy Center, Johns Hopkins University School of Medicine, Baltimore, USA

**Keywords:** Sphingosine-1-phosphate, C-reactive protein, Pneumonia, Chronic obstructive pulmonary disease

## Abstract

**Background:**

Chronic obstructive pulmonary disease (COPD) is a significant public health concern. The patients with acute exacerbations of COPD (AECOPD) and pneumonia have similar clinical presentations. The use of conventional diagnostic markers, such as complete blood count with differential and C-reactive protein (CRP), is the current mainstream method for differentiating clinically relevant pneumonia from other mimics. However, those conventional methods have suboptimal sensitivity and specificity for patients with a clinical suspicion of infection. The limitations often cause the ambiguity of the initiation of antibiotic treatment. Recently, our pilot study suggested that the patients with pneumonia have significantly higher plasma Sphingosine-1-phosphate (S1P) levels than controls. The initial findings suggest that plasma S1P is a potential biomarker for predicting prognosis in pneumonia. The aim of this study was to evaluate the value of S1P and CRP for discriminating COPD with pneumonia and AECOPD in an Emergency Department (ED) setting.

**Methods:**

Patients diagnosed with AECOPD or COPD with pneumonia were recruited from the Emergency Department of Wan Fang Hospital. The clinical data, demographics, and blood samples were collected upon ED admission. The concentration of plasma S1P was measured by ELISA.

**Results:**

Thirty-nine patients with AECOPD and 78 with COPD plus pneumonia were enrolled in this observational study. The levels of blood S1P and CRP were significantly higher in patients with COPD plus CAP compared to those in AE COPD patients. The area under the receiver operator characteristic (ROC) curve for the S1P and CRP for distinguishing between patients with COPD plus CAP and AECOPD is 0.939 (95% CI: 0.894–0.984) and 0.886 (95% CI: 0.826–0.945), whereas the combination of S1P and CRP yielded a value of 0.994 (95% CI: 0.897–1.000). By comparing with CRP or S1P, combining CRP and S1P had significantly higher AUC value for differentiating between the COPD with pneumonia group and the AECOPD group.

**Conclusions:**

Our findings suggest that S1P is a potential diagnostic biomarker in distinguishing COPD with CAP from AECOPD. Additionally, the diagnostic ability of S1P can be improved when used in combination with CRP.

## Background

Chronic obstructive pulmonary disease (COPD) is characterized by persistent respiratory symptoms and airflow limitation that is due to airway abnormalities. COPD is a leading and increasingly important cause of morbidity and mortality worldwide and is projected to be the 3rd leading cause of death by 2020 [1]. Frequent acute exacerbations of COPD (AECOPD) would increase the hospitalization and mortality rates [2]. AECOPD can be caused by bacterial infection, viral infection, allergen, and air pollution, and up to fifty percent of exacerbations are caused by bacterial infection [3]. Community-acquired pneumonia is one of the most frequent infectious causes of death worldwide[4]. Despite the advancement in treatment and diagnostic techniques, the 30-day mortality rate of pneumonia is as high as 12.1% for patients 65 years of age and older admitted to hospital [5]. Moreover, pneumonia is considered to be one of the major causes of AECOPD.

An AECOPD is a worsening of symptoms such as cough, dyspnea, and sputum production. During AECOPD, the airways resistance is rapidly increased (due to bronchospasm, mucosal edema, and sputum inspissation), which worsens expiratory flow limitation (EFL). EFL is a pathophysiological hallmark of AECOPD [6]. In addition, EFL leads to lung overinflation and further causes the gas exchange problem in the lung. Due to similar signs and symptoms, it is very challenging to differentiate bacterial and non-bacterial induced AECOPDs, especially in an Emergency Department (ED) setting. Moreover, the use of conventional diagnostic markers, such as complete blood count (CBC) with differential and C-reactive protein, is the current mainstream method for differentiating clinically relevant CAP from AECOPD. However, for patients with a clinical suspicion of infection, those conventional methods have suboptimal sensitivity and specificity [7, 8]. The limitations often cause the ambiguity of the initiation of antibiotic treatment. As a result, unnecessary use of antibiotics adversely affects patients' outcomes. Also, inappropriate antibiotic therapy increases antibiotic resistance in patients, which poses a public health problem. Current strategies to reduce antibiotic usage have included the development of biomarker-directed treatment algorithms. However, a recent study suggested that procalcitonin-guided therapy has not been effective in reducing antibiotic use [9]. Therefore, developing new diagnostic biomarkers for pneumonia may be the answer to the problems, especially for the COPD population.

C-reactive protein (CRP) has been widely used in pneumonia management [10]. CRP is a well-established biomarker of inflammation but has been considered as a non-specific marker in the pneumonia diagnosis [11], although it might have some values in defining pneumonia severity [12, 13]. Moreover, several meta-analyses have suggested that CRP performs no better than the pneumonia-specific scores in prognostic prediction [14, 15]. Sphingosine-1-phosphate (S1P) is a bioactive sphingolipid and is involved in several physiological processes, including immune responses and endothelial barrier integrity [16–21]. Additionally, our previous study suggested that S1P is a potential diagnostic, prognostic biomarker for the initial screening of patients with pneumonia [22]. In the study, we demonstrate that plasma S1P levels are significantly elevated and inversely correlated with disease severity in patients with pneumonia. Therefore, in the present study, we evaluated the value of S1P and CRP for discriminating COPD with pneumonia and AECOPD in an ED setting.

## Methods

### Patients

We conducted a prospective, single-center, observational study in the Emergency Department of Taipei Municipal Wanfang Hospital (Taipei, Taiwan) between October 2016 and February 2020. All recruited patients who presented to the ED have AECOPDs with suspected pneumonia. Before enrollment, the patients were provided with written informed consent. The inclusion criteria were: age ≧ 20 years with previously diagnosed COPD and suspected diagnosis of pneumonia as defined by the Infectious Disease Society of America (IDSA)/ American Thoracic Society (ATS) Consensus Guideline or the patient with AECOPD. For the COPD diagnosis, we followed the GOLD guideline. The follow-up or the admitting pulmonologists provided the final diagnosis. The diagnosis criteria of pneumonia were based on the American Thoracic Society/Infectious Diseases Society of America Community-Acquired Pneumonia Guideline. The patient who had pneumonia in the previous 30 days, active tuberculosis, aspiration pneumonia, immune-deficiency (due to HIV infection, prior transplantation, immunosuppressive therapy, or neoplasm), or the pregnancy was excluded from our study.

The peripheral blood was collected from the patients presenting at the emergency department (ED) of Wan Fang Hospital. The following parameters were recorded for each participant: sex, age, body weight, body temperature, vital signs at the ED, and clinical characteristics of the disease. The laboratory testing includes baseline analyses and CRP. For the patients with pneumonia, the pneumonia severity index (PSI) [23] and CURB-65 [24] were also calculated.

### Measurement of sphingosine-1-phosphate

The peripheral blood samples were stored in tubes containing EDTA. The samples were centrifuged at 2500×*g* for 10 min, and the upper plasma layers were collected. The samples were then frozen at – 80 °C for storage. A commercially available enzyme-linked immunosorbent assay (ELISA) kit (MyBiosource) was used for plasma S1P concentration measurements.

### Statistical analysis

Statistical analysis was performed with the R 3.6.1 software (R Foundation for Statistical Computing, Vienna, Austria). Based on the previous study [25] and our initial pilot study, we assumed a 20 ng/ml difference in serum S1P with a standard deviation of 30 ng/ml. A sample size of 44 per group (88 total) will be sufficient to detect a between-group difference, assuming a power of 90%, alpha of 5%. The data were presented median and interquartile range or mean and standard deviation (SD). The categorical variables are expressed as counts or percentages. Youden indexes were used to determine the optimal cut-off value on a receiver operating characteristic (ROC) curve. In terms of areas under two ROC curves comparison, the empirical (nonparametric) methods were used [26]. The degree of association between variables was measured by the Spearman rank correlation test. In the comparison between groups, the Mann–Whitney U-test was used. Statistical tests were two-sided, and p < 0.05 was accepted as statistically significant.

## Results

### Characteristics of the Study Population

A total of 127 patients (AECOPD: 49 and COPD with pneumonia: 78) were recruited in this study. Based on the GOLD guidelines, the AECOPD severity of the patients in our population is defined as severe. The baseline characteristics of the patients are shown in Table 1. No difference was found between the two groups in age, gender, hospital mortality, and comorbidities. However, COPD patients with pneumonia had higher hospital admission rates and ICU admission rates. The pneumonia severity index and CURB-65 were used for pneumonia severity assessment.

**Table 1:** Demographic and baseline characteristics of patients and disease status

	AECOPD	COPD with CAP	p-value
Participants, n	49	78	–
Mean age (SD), year	72.6 (15.7)	76.3 (12.4)	0.15
Male/female (%), n	30/19 (61.2%)	46/32 (58.9%)	0.97
Admission (%), n	39 (79.6%)	75 (96.2%)	< 0.01
ICU admission (%), n	2 (4.1%)	14 (17.9%)	< 0.05
Hospital mortality (%), n	1 (2.0%)	5 (6.4%)	0.41
Comorbidities			
Hypertension (%), n	23 (46.9%)	35 (44.9%)	0.96
Diabetes mellitus (%), n	16 (32.7%)	20 (25.6%)	0.69
PSI			
≦90	–	18 (23.0%)	–
91–130	–	45 (57.7%)	–
> 130	–	15 (19.3%)	–
CURB-65			
0–1	–	42 (53.8%)	–
2	–	24 (30.8%)	–
3–5	–	12 (15.4%)	–

### Biomarkers levels

**Fig. 1:**
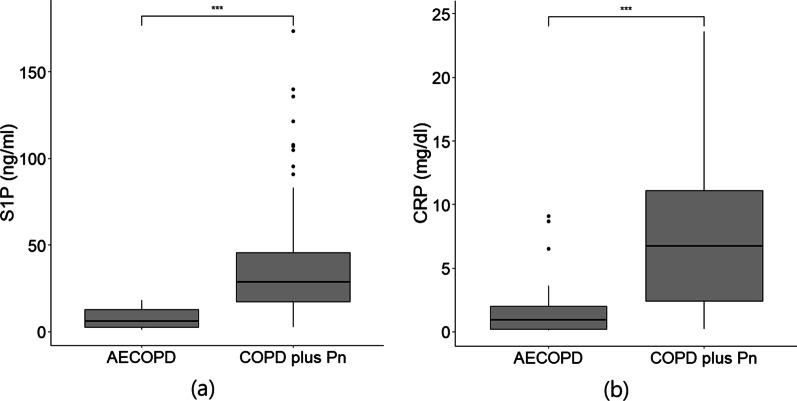
The distribution of plasma **a** S1P levels and **b** CRP levels in patients with AECOPD or COPD plus pneumonia

Levels of S1P ranged from 1.1 to 173.7 ng/ml. S1P conxentration was significantly higher in COPD with pneuomina group (Median: 28.8, IQR: 45.6–17.2) compated to AECOPD group (Median: 5.9, IQR: 13.0–2.7) (p < 0.001; Fig. 1a). Concentrations of CRP ranged from 0.1 to 23.6 mg/dl. The COPD patient with pneuomina (Median: 6.7, IQR: 23.6–2.4) presented significantly higher CRP levels than those in patient with AECOPD (Median: 0.9, IQR: 9.1–0.2) (p < 0.001; Fig. 1b). In the univariate logistic regression analysis, S1P concentration was predictive of pneumonia with odds ratio of 1.27 (95% CI: 1.17–1.41; p < 0.0001) and CRP levels was predictive of pneumonia with odds ratio of 1.59 (95% CI: 1.35–1.99, 1.43–3.51 p < 0.0001). In terms of the multivariate logistic regression analysis, S1P (OR: 2.00, 95% IC: p < 0.005) and CRP (OR: 2.68, 95% IC: 1.71–5.77, p < 0.001) were significant factors in differentiating between the COPD with pneuomina group and the AECOPD group. Moreovere, there was significant correlations between the level of S1P and CRP (rho = 0.312, p < 0.001). For the full panel of the univariate and multivariate logistic regression analysis, the results were shown in Table 2.

**Table 2:** Diagnostic performance of S1P level and CRP level for pneumonia in univariate and multivariate logistic regression analysis

	Univariate analysis			Multivariate analysis		
Biomarkers	OR	CI (95%)	p-value	OR	CI (95%)	p-value
S1P	1.27	1.17–1.41	> 0.0001	2.00	1.43–3.51	> 0.005
CRP	1.59	1.35 -1.99	> 0.0001	2.68	1.71–5.77	> 0.001

### Diagnostic performance of the biomarkers

**Fig. 2:**
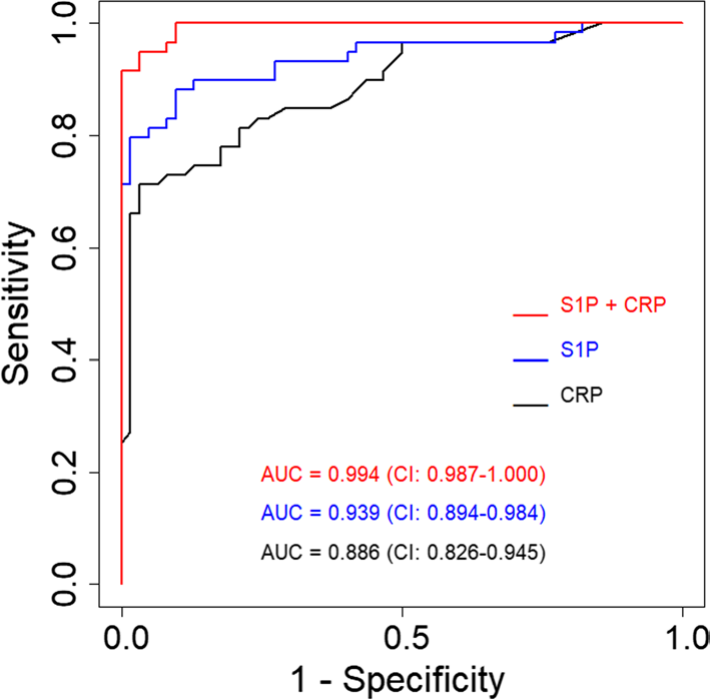
Receiver operating characteristic curves for S1P and CRP in blood for distinguishing between patients with AECOPD and COPD plus pneumonia

We assessed the discriminatory power of the biomarkers in differentiating between the COPD with pneumonia group and the AECOPD group by using ROC curve analysis. In the ROC analysis, the area under the curve in roc curve was 0.939 (95% CI: 0.894–0.984) for S1P and 0.886 (95% CI: 0.826–0.945) for CRP (Fig. 2). For S1P, a cut-off value of 16.9 ng/ml yielded a sensitivity of 76.92% and specificity of 97.96. For CRP, A cut-off value of 3.5 mg/dl yielded a sensitivity of 71.79% and specificity of 91.84% (Table 3). There were no statistically significant differences between S1P and CRP in separating the COPD with pneumonia group from the AECOPD group (p = 0.254). By combining S1P and CRP, the area under the curve in ROC curve increased to 0.994 (95% CI: 0.897–1.000) (Fig. 2). By comparing with CRP or S1P, combining CRP and S1P had significantly higher AUC value for differentiating between the COPD with pneumonia group and the AECOPD group (p < 0.001 and p < 0.005, respectively).

**Table 3:** Comparing different methods for predicting patient diagnosis status

	Sensitivity (%)	Specificity (%)	PPV (%)	NPV (%)
S1P > 16.9	76.92	97.96	98.36	72.73
CRP > 3.5	71.79	91.84	93.33	67.16
S1P > 16.9 or CRP > 3.5	98.72	89.80	93.90	97.78

*PPV* positive predictive value, *NPV* negative predictive value

## Discussion

Since acute exacerbations and pneumonia present with similar signs and symptoms in the patient with COPD, it is challenging to distinguish them early in an emergency room setting. The problem not only results in misdiagnosis but also inappropriate usage of antibiotics [27, 28]. The Global Initiative for Chronic Obstructive Lung Disease (GOLD) strategy documents suggest that antibiotics usage should be based on clinical signs of infection, blood CRP level, and blood procalcitonin level. However, in clinical use, the method is still suboptimal. Therefore, we tried to develop a new strategy for differentiating pneumonia from acute exacerbation in the COPD population. This study demonstrates that COPD patients with pneumonia presented significantly higher blood CRP lever and higher blood S1P compared to patients with AECOPD. There was a weak correlation (rho = 0.312) between S1P and CRP concentrations, and both biomarkers have acceptable diagnostic accuracy (measured as AUC). Further, the multivariate logistic regression analysis suggested both S1P and CRP are independent predictors for COPD patients with pneumonia. Moreover, by combining the biomarkers, diagnostic accuracy was significantly increased with excellent sensitivity and specificity.

CRP is an acute-phase protein synthesized in the liver. The serum CRP concentration increases during infections, especially in bacterial infections. A previous study suggested CRP is the most selective biomarker in the diagnosis of AECOPD with insufficient specificity and sensitivity [29]. The following research demonstrated that, unlike procalcitonin, CRP is associated with airway bacterial presence and the treatment effect of antibiotics in AECOPD patients increases with higher values of CRP [30]. Current researches also included CRP as a biomarker for pneumonia in COPD patients, and they reported acceptable diagnostic accuracy (AUC from 0.63 to 0.84) [31, 32]. S1P has been suggested involved in acute lung injury and sepsis [33–35]. A previous study also indicated that lower serum-S1P levels are associated with severe sepsis and septic shock [25]. Moreover, our previous study demonstrated that blood S1P concentrations are inversely associated with pneumonia severity [22]. Therefore, when we use only S1P for discriminating between pneumonia and acute exacerbation in COPD patients, the patient with severe pneumonia would easily be missed. Due to severe bacterial infection status, the CRP level is usually very high in patients with severe pneumonia. As a result, by combining S1P and CRP, we obtained a significantly higher diagnostic accuracy for the diagnosis of pneumonia in a patient with COPD in the emergency room setting.

There are several studies trying to identify new biomarkers for distinguishing between pneumonia and AECOPD. Pizzini et al. demonstrated that the level of pteridine neopterin (NPT), a marker for immune system activation, is higher in pneumonia patients compared to AECOPD patients. They further suggested utilizing CRP/NPT ratio in serum to discriminate pneumonia from AECOPD in COPD patients [36]. Bertrams et al. discovered a panel of genes in PBMCs that were differentially expressed between pneumonia and AECOPD patients and found several microRNAs, which separated pneumonia and AECOPD. They further identified HNF4A, MCC, and MUC1 as the most important discriminatory markers [37]. Recently, small extracellular vesicles (sEVs) were also suggested to be used as biomarkers for discriminating between CAP and AECOPD [38]. The sEVs are membrane-contained released from most cell types and can be found in blood [39]. In that study, the authors identified a panel of surface proteins of plasma sEVs as biomarkers for the differentiation of pneumonia and AECOPD. Here, in our study, we identified a new potential biomarker, S1P, for the diagnosis of pneumonia in COPD patients.

Our study has a few limitations. First, this is a single-center with a relatively small sample size study, and the patients were heterogeneous in terms of clinical severity of their AECOPD or pneumonia. Our research focus of the pilot study was to discover the biomarkers for distinguishing between pneumonia and AECOPD. The study lacks an independent validation cohort. Further studies with larger patient numbers and an independent validation cohort will be necessary to confirm our observations. Also, the information of the detailed treatment and clinical history was not obtained in the study. The S1P and CRP were measured only once at a single time point during screening. Thus, the information of reproducibility and concentration change during the time course of the biomarkers is missing. Furthermore, in terms of patient selection, the participants were recruited from the emergency room and usually had a more serious exacerbation episode. As a result, the results cannot be extrapolated to the COPD population as a whole.

## Conclusions

The blood S1P was significantly higher in patients with COPD plus CAP than those in patients with AECOPD. The S1P also has a reasonable specificity and positive predictive value in the diagnosis of pneumonia in a patient with COPD. Thus, our findings suggest that S1P is a potential diagnostic biomarker for CAP, especially in distinguishing COPD with CAP from AE COPD. Moreover, the diagnostic ability can be improved when used in combination with CRP.

## Data Availability

The datasets used and/or analyzed during the current study are available from the corresponding author on reasonable request.

## Abbreviations

COPD: Chronic obstructive pulmonary disease
AECOPD: Acute exacerbations of COPD
CRP: C-reactive protein
S1P: Sphingosine-1-phosphate
ED: Emergency Department
